# Research on Microstructure and Properties of Ultrasonic Welded Large-Diameter Aluminum Wire/Cu (Ni-Plated Copper) Terminal Joints

**DOI:** 10.3390/ma19091749

**Published:** 2026-04-24

**Authors:** Yi Bu, Ye Zhao, Shupeng Zhao, Yanrong Ni, Lipeng Yan

**Affiliations:** 1 Wuxi Xinhongye Wire & Cable Co., Ltd., Wuxi 214101, China; 2 School of Cable Engineering, Henan Institute of Technology, Xinxiang 453003, China; 3 College of Material Science and Engineering, Henan Institute of Technology, Xinxiang 453003, China

**Keywords:** ultrasonic welding, Al wires, Cu terminal, nickel-plated copper terminal, microstructure

## Abstract

In this study, the microstructure and mechanical properties of ultrasonic welded joints between large-diameter aluminum wire and Cu (Ni-plated copper) terminals were systematically investigated, to reveal the underlying fracture mechanisms. The cross-sectional morphology, interfacial microstructure, and mechanical properties of the two types of welded joints are investigated. The results indicate that ultrasonic welding produces well-structured Al-Cu and Al-Ni joints. Under the same welding process parameters, the Al-Cu joint exhibits many pores, while the Al-Ni joint has no pores in its microstructure. The interfacial region of the Al-Cu joint presents various morphologies, such as flat bonding, interlocking, and eddy current patterns, whereas the Al-Ni joint interface is flat. No significant atomic diffusion phenomenon occurs between the interfaces of the two types of joints. The tensile strength of the Al-Cu joint is 53 MPa, with fracture modes including ductile fracture and brittle fracture, whereas the tensile strength of the Al-Ni joint is 50 MPa, with a failure mode of pull-out fracture. In working conditions requiring ultrasonic welding of aluminum and copper, nickel-plated copper can be used as a substitute for copper to prevent electrochemical corrosion between aluminum and copper.

## 1. Introduction

Wire harness and terminal connection products are crucial components of cable assemblies in the manufacturing of new energy vehicles, providing reliable electrical connections and mechanical support for the vehicles, ensuring the stability and reliability of signal transmission and power supply [1,2,3]. Copper, with its excellent electrical and thermal conductivity, is an ideal material for terminals and wire harnesses. However, copper’s high density and cost hinder the current pursuit of lightweight and affordable vehicles. Aluminum, with its low density, good conductivity, and low cost, offers advantages. When the diameter of aluminum wire harness is increased to achieve the same resistivity as the copper wire, the product weight can still be reduced by 50%. Therefore, replacing copper wire with aluminum wire will help to reach the goals of lightweight vehicles and cost reduction. Due to significant differences in physical properties, such as melting point and thermal conductivity between copper and aluminum, traditional fusion welding tends to form thick and brittle intermetallic compounds, such as Al_2_Cu and Al_4_Cu_9_, at the interface, leading to deteriorated mechanical properties and conductivity of the joint [4,5].

Ultrasonic welding is a solid-phase joining technology that can avoid the formation of a liquid phase at the joint interface and achieve material joining through mechanisms such as deformation and atomic diffusion. Therefore, the ultrasonic welding technology has broad application prospects in fields such as automobile manufacturing, aerospace industry, and electronic equipment manufacturing [6,7]. However, aluminum/copper ultrasonic welding can also produce intermetallic compounds. Liu et al. [8] discovered copper/aluminum intermetallic compounds (IMCs) when ultrasonically welding 0.8 mm thick copper and aluminum plates, and their research showed that an additional heat source promoted the welding process; Li et al. [9] found IMCs during ultrasonic welding of copper/aluminum plates and believed that as the welding pressure increased, the thickness of IMCs first increased and then decreased. In contrast, metallic nickel has good corrosion and wear resistance. In air, the surface of nickel undergoes oxidation reactions to form a dense oxide layer, which is commonly used as a functional coating on other metal surfaces to improve the wear resistance and oxidation resistance of the protected metal [10].

In this article, the microstructure and properties of the joints by ultrasonic welding aluminum wire harnesses/pure copper terminals and aluminum wire harnesses/nickel-plated copper terminals were researched. Optical microscopy, scanning electron microscopy, and tensile testing machines are utilized to test the macroscopic morphology, interfacial microstructure, fracture morphology, and tensile load of the joints. The microstructure and properties of the two ultrasonic welding joints are studied to provide technical theoretical support for meeting engineering application requirements.

## 2. Materials and Methods

**Figure 1:**
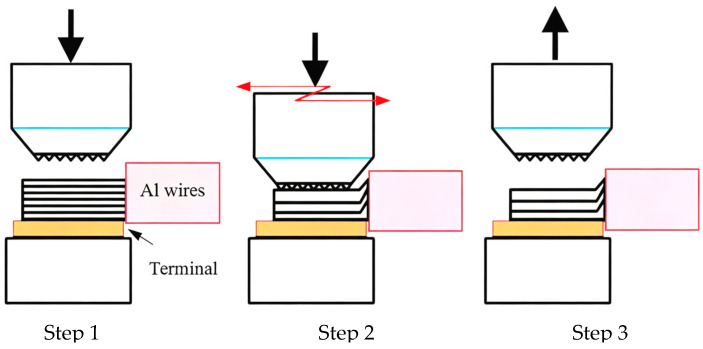
Schematic diagram of Al wires/terminal ultrasonic welding process.

The ultrasonic welding steps involved in the experiment are shown in Figure 1. First, the terminal is placed above the anvil, and the aluminum wire harness is lapped with the terminal. The welding head moves downwards at a set pressure and presses onto the wire. Then, the welding head vibrates at the set frequency and amplitude in a high-frequency manner, with the vibration direction parallel to the axial direction of the aluminum wire, and the joint connection is achieved through the generation of frictional heat between the workpieces. Finally, the welding head moves upwards, and the welding process ends. The base materials used in the experiment are industrial pure aluminum (1060) wires, commercial T2 pure copper terminals, and nickel-plated copper terminals. The compositions of the 1060 Al wires and T2 Cu are shown in Table 1 and Table 2, respectively.

**Figure 2:**
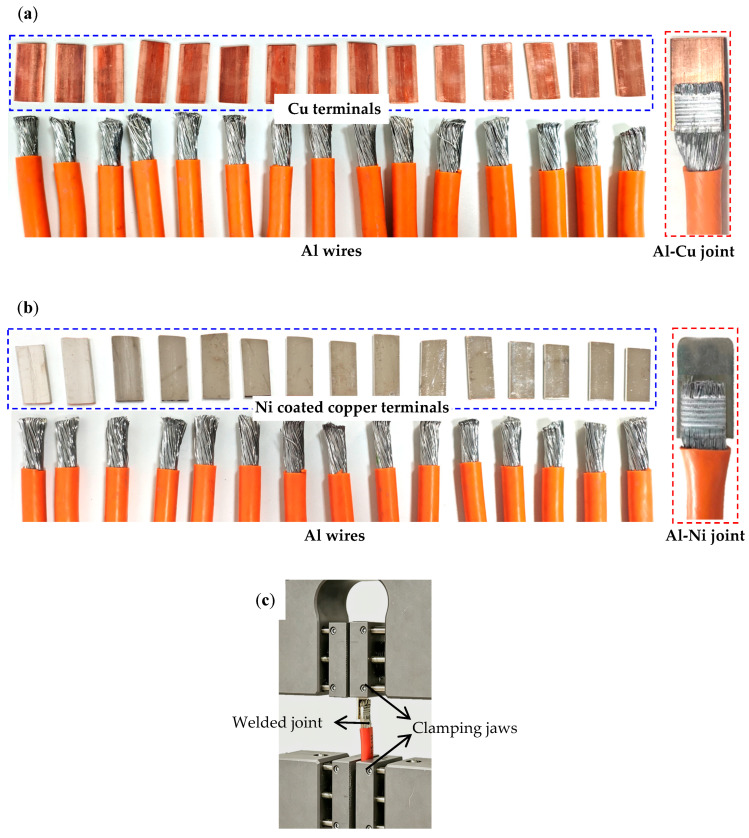
(**a**) Cu terminals, Al wires and Al-Cu joint; (**b**) Ni-coated copper terminals, Al wires and Al-Ni joint; (**c**) tensile shear test setup.

The cross-sectional area of the aluminum wire harness is 50 mm^2^, and the diameter of a single wire is 0.5 mm; the terminal size is 35 mm × 16 mm × 2 mm, and the welding size is 16 mm × 14 mm. The surface roughness of Cu terminals and Ni-plated Cu terminals is 0.2. A GMX-20MA (Emerson, MO, USA) ultrasonic welder is used, with a frequency of 20 kHz, a welding pressure of 0.5 MPa, a welding amplitude of 54 μm, and a welding time of 2 s. The aluminum wire harness, Cu terminals, Ni-coated copper terminals, and ultrasonic welding joints are shown in Figure 2. The number of Al-Cu and Al-Ni welded joints is 15, respectively. The macroscopic morphology of the joint section was observed using an PME-3 (Olympus, Tokyo, Japan) optical microscope (OM); the microstructure of the interface was examined using a JSM-7800F (JEOL, Tokyo, Japan) scanning electron microscope (SEM) equipped with an Octane Plus of EDAX energy dispersive spectrometer (EDS); the welded joint was subjected to tensile shear testing using a AG-1250KN (Shimadzu, Kyoto, Japan) universal tensile testing machine at a tensile speed of 10 mm/min. The number of replicates for each test is 15 respectively and the tensile shear test is depicted in Figure 2c.

## 3. Results and Discussion

### 3.1. Macroscopic Morphology of Welding Joint Cross-Section

**Figure 3:**
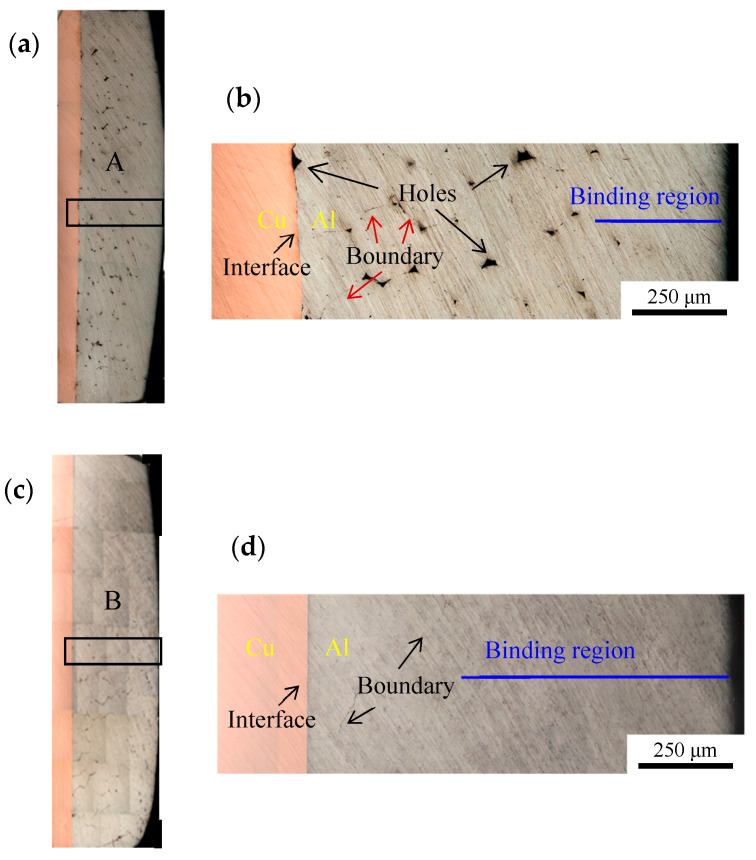
Cross-section of the joints. (**a**) Morphology of Al-Cu joint. (**b**) Enlarged drawing of the line box A. (**c**) Morphology of Al-Ni joint. (**d**) Enlarged drawing of the line box B.

Figure 3a shows the cross-sectional morphology of the ultrasonically welded aluminum wires/copper terminal joint. By zooming in area A marked with a wire frame in Figure 3a, Figure 3b is obtained. In the top area, there are few holes, indicating the formation of metallurgical bonding between the wire harnesses, and in the middle area, there are many black holes and wire harness boundary lines, as indicated by the arrows. The interface between the Al wire harness and the terminal is not straight, exhibiting a distinct wavy structure. There are also a small number of pores at the interface between the aluminum wire harness and the terminal, indicating that the joint microstructure has not achieved complete bonding. The figure shows the presence of various connection states in the joint microstructure, including pores, line connections, and metallurgical bonding. This is because the top part is close to the tool head, and under the vertical pressure, the aluminum wire harness transitions from a “stick-slip” state to a viscous state, thus forming a tight connection between the wires. The vibration friction force propagates from top to bottom, converting a large amount of kinetic energy into thermal energy, which binds the aluminum wire harness to the copper terminal [11]. However, during the process of reaching the bottom terminal, the amplitude gradually decreases, and the thermal energy converted from kinetic energy gradually decreases. These thermal energies are not sufficient to support metallurgical bonding between the wires, resulting in many holes.

Figure 3c depicts the cross-sectional morphology of the ultrasonically welded aluminum wire harness/nickel-plated terminal joint. By zooming in the wire frame area B in Figure 3c, Figure 3d is obtained. The figure reveals that no significant holes is observed in the structure, and only a small number of boundary lines are found. The nickel plating layer between the wire harness and the terminal is straight without any noticeable deformation. This suggests that a metallurgical bond has been formed between the aluminum wire harnesses. This is attributed to the high hardness of nickel. During the ultrasonic welding, the aluminum wire harnesses are simultaneously subjected to the forces exerted by the welding head and the nickel layer, increasing the contact area between the aluminum wire harnesses. These contact areas serve as potential friction and deformation bonding zones. The welding amplitude induces relative sliding and plastic deformation between the aluminum wires, aluminum and nickel. The friction and plastic deformation of the materials generate a large amount of heat, which increases the temperature of the base material, providing a driving force for the diffusion of atoms at the interface, leading to metallurgical bonding between aluminum–aluminum and aluminum–nickel interfaces [12]. Consequently, the effective bonding length between aluminum and nickel in the aluminum wire harness/nickel-plated terminal joint structure significantly increases, while the aluminum–aluminum gap decreases.

### 3.2. Interfacial Microstructural Characteristics of the Joints

**Figure 4:**
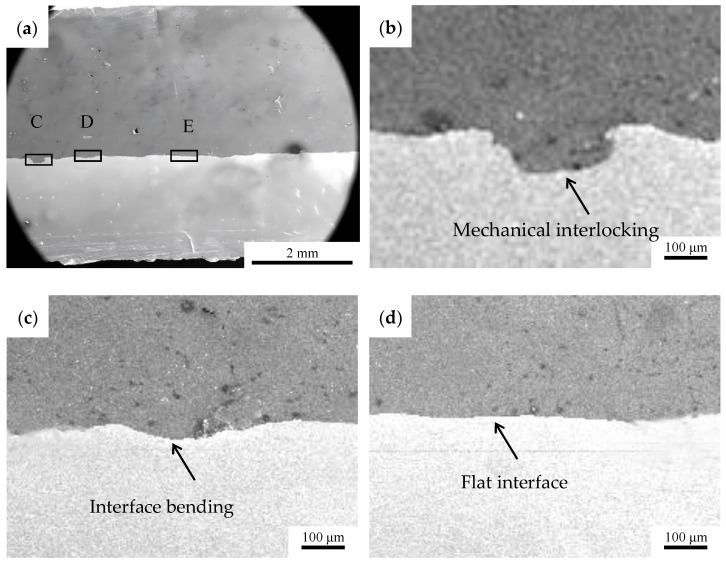
(**a**) Interface SEM morphology of the Al–Cu joint. (**b**) Enlarged drawing of the line box C in Figure 4a. (**c**) Enlarged drawing of the line box D. (**d**) Enlarged drawing of the line box E.

Figure 4a depicts the interfacial morphology of the ultrasonic welding joint between aluminum wire harness and pure copper terminal. The figure reveals that the Al–Cu contact interface is not flatly bonded, but exhibits a concave–convex morphology. Figure 4b–d show an enlarged interfacial view within the line boxed area in Figure 4a. The figures display complex and diverse microstructural morphologies, including flat bonding, interlocking, and eddy current-like plastic flow layers. From Figure 4b, it can be observed that there is a distinct eddy current-like plastic deformation zone with a height of approximately 90 µm at the joint, but almost no voids or cracks are found, indicating high welding quality [13,14,15]. Figure 4c,d demonstrate that the interface exhibits curved and wavy morphologies. The above indicates that the interface is tightly connected, forming a mechanical self-locking. This is because under the interaction of high-frequency vibration and pressure, both aluminum and copper undergo uneven plastic deformation, leading to uneven distribution of internal stress near the welding interface. The softened metal undergoes plastic flow under the influence of uneven internal stress [16].

**Figure 5:**
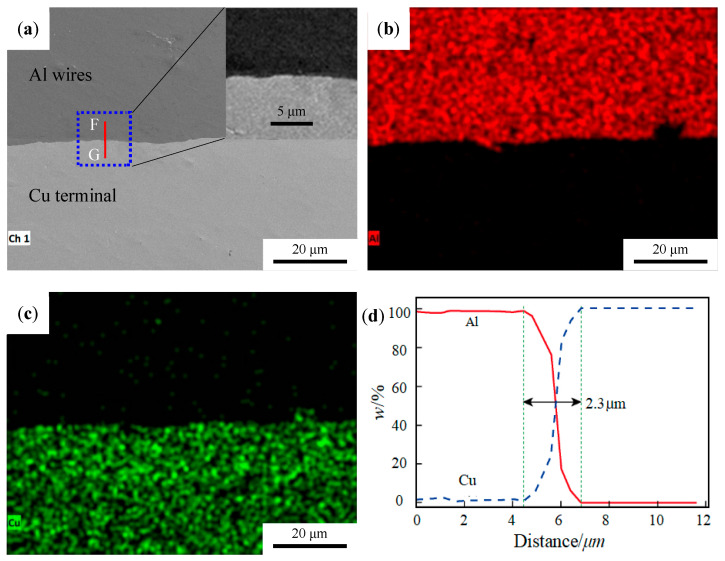
Interface map of Al–Cu joint. (**a**) SEM morphology of the Al–Ni joint. (**b**,**c**) Element mapping of the area. (**d**) EDS line scan corresponding to F→G in Figure 5a.

Figure 5a shows the microstructure analysis of welding interface and element distribution map of the interface. Figure 5b,c show the elemental mapping, investigating the elemental distribution of the interface. And no obvious interlayer is found on the interface. Figure 5d is the EDS line scan map. The F→G line scan in the figure reveals that the atomic transition distance is approximately 2.3 μm, and there is no platform transition area geometrically, indicating that no intermediate phase, such as Al_2_Cu phase, has been formed at the interface. This is because the welding time during the ultrasonic welding is short and the peak welding temperature is low (not reaching the melting point of the base material), which is insufficient to drive the formation of intermediate phases at the reaction interface.

**Figure 6:**
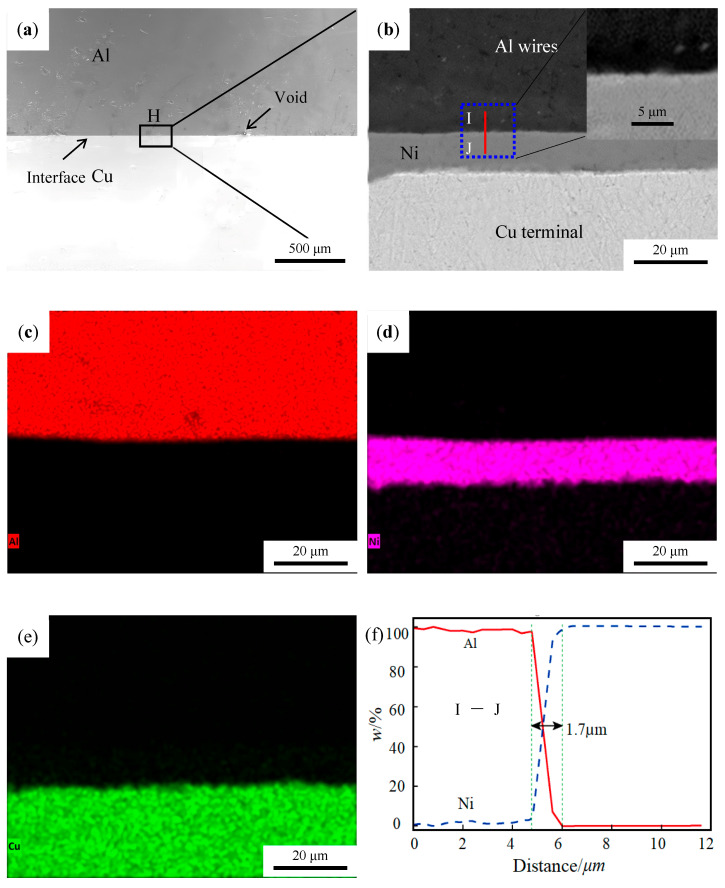
Interface map of Al–Ni joint. (**a**) SEM morphology of the Al–Ni joint. (**b**) Enlarged drawing of the line box in Figure 6a. (**c**–**e**) Element mapping of the area. (**f**) EDS line scan corresponding to I→J in Figure 6b.

Figure 6a is an SEM image of the cross-sectional view of the ultrasonic welding joint of aluminum wire/nickel-plated copper terminal. The image shows that the Al–Ni interface is straight and has not bent. Under the interaction of high-frequency vibration and pressure, aluminum undergoes significant plastic deformation, but due to the high hardness of the nickel layer, these forces are not transmitted into the copper terminal, resulting in a straight bonding state at the Al–Ni interface. An enlarged analysis of the wire frame area H in Figure 6a is shown in Figure 6b. The image reveals a nickel layer approximately 10 µm thick, with a straight Al–Ni interface and no obvious intermediate layer. Figure 6c–e show an analysis of the elemental mapping, investigating the elemental distribution of the interface. The images indicate that no obvious intermetallic compounds are produced between Al and Ni.

Figure 6f shows the results of EDS line scan (I→J) applied to the cross-sectional surface of the joint. The figure shows that the thickness of the atomic transition layer at the interface is about 1.7 μm, which indicates the formation of metallurgical bonding between aluminum and nickel. The Al element decreases smoothly from 100% to 0%, and Ni element increases smoothly from 0% to 100%. Furthermore, no obvious platform is formed at the Al-Ni interface. Therefore, it can be inferred that no stable intermetallic compound is formed at the Al-Ni interface, and the two materials achieve effective connection mainly through mechanical interlocking [17].

### 3.3. Mechanical Properties of the Joints

**Figure 7:**
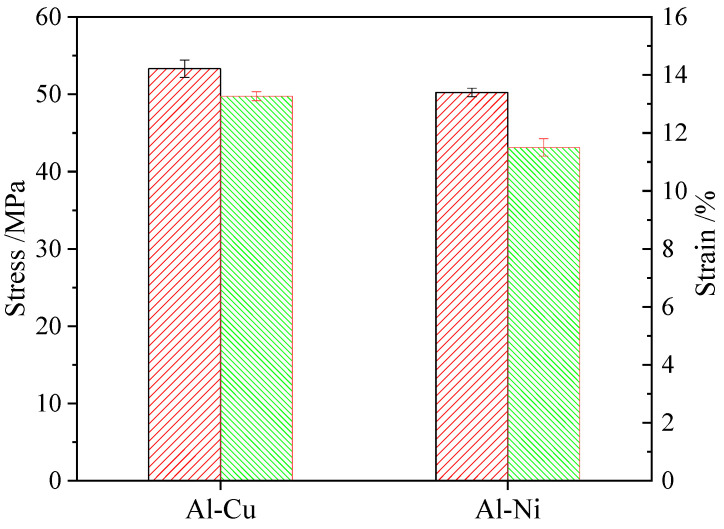
Stress–strain histogram of Al-Cu joint and Al-Ni joint.

Tensile shear strength of 15 specimens were tested respectively, as shown in Table 3. Figure 7 illustrates the tensile stress–strain relationship of ultrasonically welded joints between Al-Cu and Al-Ni. The figure demonstrates that, under identical welding parameters, both ultrasonically welded joints exhibit comparable shear strength, with the Al-Cu joint achieving a shear strength of 53 MPa and the Al-Ni joint reaching 50 MPa. This suggests that, in working conditions requiring ultrasonic welding of Al and Cu, Ni-plated copper can be used as a substitute for copper to prevent electrochemical corrosion between Al and Cu.

### 3.4. Fracture Morphology

**Figure 8:**
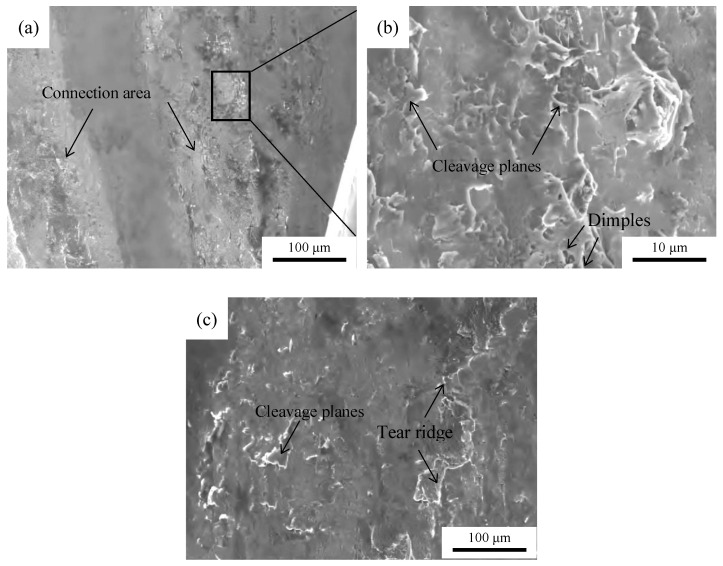
Fracture morphology. (**a**) Al-Cu joint. (**b**) Enlarged drawing of the line box in Figure 8a. (**c**) Al-Ni joint.

Figure 8a presents an SEM image of the tensile fracture for the Al/Cu joint. The image reveals that the joint fracture occurs at the copper–aluminum interface, with distinct unconnected and connected areas present on the copper terminal. The high-magnification observation of the connected area is shown in Figure 8b. The presence of numerous cleavage facets and a small number of shallow dimples on the interface indicates that the fracture mode of the Al/Cu joint is a brittle–ductile mixed fracture. Figure 8c shows an SEM image of the tensile fracture for the Al/Ni joint. The image demonstrates that the joint fracture occurs at the Al/Ni interface, with almost the entire Ni terminal interface being a connected area, exhibiting typical cleavage facets and tearing ridge morphologies, indicating that the failure mode of the joint is a pull-out fracture [18].

## 4. Conclusions

(1) Ultrasonic welding of Al-Cu and Al-Ni both achieved well-structured joints. Under the same welding process parameters, there are many holes between the aluminum wires and at the interface in the Al-Cu joint, whereas there are no pores between the aluminum wires and at the interface in the Al-Ni joint.

(2) The interface zone of ultrasonically welded Al-Cu joints exhibits various morphologies such as flat bonding, interlocking, and eddy current-like plastic flow layers, with no evident atomic diffusion between interfaces. Meanwhile, the interface zone of Al-Ni joints presents a flat bonding morphology, without plastic deformation, and no significant atomic diffusion between interfaces. 

(3) The tensile strength of ultrasonically welded Al-Cu joints is 53 MPa, with a fracture mode consisting of both ductile and brittle fractures. Conversely, the tensile strength of Al-Ni joints is 50 MPa, and the failure mode is pull-out fracture. In working conditions requiring ultrasonic welding of aluminum and copper, nickel-plated copper can be used as a substitute for copper to prevent electrochemical corrosion between aluminum and copper.

## Figures and Tables

**Table 1:** Chemical element composition of 1060 aluminum (%).

Al	Fe	Si	Zn	Ti	Mn	Cu	Mg
99.7	0.30	0.20	0.05	0.03	0.03	0.05	0.03

**Table 2:** Chemical element composition of T2 copper (%).

Cu	Fe	Pb	S	Bi	Sb	As
99.9	0.005	0.005	0.005	0.001	0.002	0.002

**Table 3:** Tensile shear strength of Al-Cu joint and Al-Ni joint.

	1#	2#	3#	4#	5#	6#	7#	8#	9#	10#	11#	12#	13#	14#	15#
Al-Cu joint	52.8	53.4	54.1	51.9	52.6	52.3	53.4	53.2	54.3	53.6	52.6	53.5	53.2	52.1	52.0
Al-Ni joint	49.8	50.4	51.3	48.6	49.4	49.5	50.6	50.0	51.4	50.5	49.7	50.4	50.3	49.1	48.9

## Data Availability

The original contributions presented in this study are included in the article. Further inquiries can be directed to the corresponding author.
